# Alterations in the Immune Response in Individuals with Latent Tuberculosis Infection

**DOI:** 10.3390/pathogens15010014

**Published:** 2025-12-22

**Authors:** Anna Starshinova, Adilya Sabirova, Igor Kudryavtsev, Artem Rubinstein, Arthur Aquino, Leonid P. Churilov, Ekaterina Belyaeva, Anastasia Kulpina, Raul A. Sharipov, Ravil K. Tukfatullin, Nikolay Nikolenko, Dmitry Kudlay

**Affiliations:** 1 Department of Mathematics and Computer Science, Saint Petersburg State University, 199034 Saint Petersburg, Russia; adilyasabirova@mail.ru (A.S.); asya.starshinova@mail.ru (A.K.); 2 Almazov National Medical Research Center of the Ministry of Health of the Russian Federation, 2, Akkuratov Str., 197341 Saint Petersburg, Russia; 3 Medicine Department, Bashkir State Medical University, 450008 Ufa, Russia; 4 Institute of Experimental Medicine, 197376 Saint Petersburg, Russia; 5 Department of Medicine, Saint Petersburg State University, 199034 Saint Petersburg, Russia; 6 The Moscow Research and Clinical Center for Tuberculosis Control of the Moscow Government Department of Health, 127006 Moscow, Russia; 7 Department of Pharmacology, Institute of Pharmacy, Sechenov University, 119991 Moscow, Russia; 8 NRC Institute of Immunology FMBA of Russia, 115522 Moscow, Russia; 9 Department of Bioengineering and Bioinformatics, Lomonosov Moscow State University, 119991 Moscow, Russia

**Keywords:** latent tuberculosis infection, preclinical stage, *Mycobacterium tuberculosis*, immune biomarkers, interferon signature, extracellular vesicles, transcriptomics, immunodiagnostics, PET/CT imaging, multidrug-resistant tuberculosis

## Abstract

Latent tuberculosis infection (LTBI) represents a biologically active yet clinically asymptomatic stage of *Mycobacterium tuberculosis* (Mtb) persistence. This condition is characterized by subtle immunometabolic alterations reflecting the host–pathogen equilibrium. Understanding the mechanisms and biomarkers associated with the preclinical phase of LTBI is crucial for preventing progression to active tuberculosis (ATB). Recent advances have identified multiple immunological, transcriptomic, metabolic, and imaging-based approaches that enable stratification of individuals at increased risk of LTBI reactivation. Quantitative assays such as IGRA, multiplex and T-cell activation marker (TAM) tests, as well as interferon-related transcriptional signatures, demonstrate predictive potential when combined with functional assays (MGIA) and metabolic imaging (PET/CT). Experimental primate models faithfully reproduce the spectrum from latency to reactivation, allowing for validation of biomarkers and vaccine or immunomodulatory strategies. The review also highlights the particular challenges of multidrug-resistant LTBI (MDR-LTBI), where standard chemoprophylaxis is less effective and immune control plays a decisive role. The preclinical phase of LTBI constitutes a key point in the TB control cascade. Integrating immunological, transcriptomic, and radiological data into risk-based screening algorithms could substantially improve early detection and targeted prevention. Translating research-derived signatures into clinically applicable, standardized, and cost-effective diagnostic tools requires coordinated international efforts, technological transfer, and policy-level support to reduce TB reactivation and transmission, including MDR-TB.

## 1. Introduction

Latent tuberculosis infection (LTBI) remains one of the most significant challenges in modern phthisiology, as it represents a potential reservoir for the subsequent development of active tuberculosis (ATB) and continues to pose difficulties in diagnosis and prevention. As is known, the Russian Federation (RF) was removed from the list of high tuberculosis burden countries as early as 2022 [1]. According to the *WHO Global Tuberculosis Report 2024*, the tuberculosis incidence rate in Russia in 2023 was 38 per 100,000 population. Between 2015 and 2023, this indicator decreased by 43%, while the total number of tuberculosis-related deaths declined by 58% over the same period [2].

The COVID-19 pandemic had a substantial impact on tuberculosis incidence trends, complicating its diagnosis and control, particularly among the most vulnerable patient groups. Individuals with LTBI are characterized by an increased risk of developing active disease, especially in the context of post-COVID immune alterations and the presence of post-COVID syndrome [3,4].

According to the World Health Organization (WHO) [2], in 2023, 10.8 million people developed tuberculosis, equivalent to 134 cases per 100,000 population. A consistent increase in the number of new cases was observed between 2021 and 2023: 10.4 million in 2021, 10.7 million in 2022, and 10.8 million in 2023. Analysis of global trends indicates that the COVID-19 pandemic contributed to an increase in tuberculosis incidence, partly due to the reactivation of latent infection among individuals who did not receive preventive therapy, as well as among those recently exposed to infectious patients or those with weakened immune systems [5].

In the long-term perspective, a pronounced decline has also been observed: over the past 13 years (2011–2024), tuberculosis incidence in the Russian Federation has nearly halved—from 49.4 to 26.5 cases per 100,000 population. In 2024, 38,753 newly diagnosed cases were reported, representing a 9.65% decrease compared with 2023 [6].

Thus, despite the global increase in tuberculosis incidence associated with the consequences of the COVID-19 pandemic, Russia continues to demonstrate stable positive trends in reducing both morbidity and mortality. Nevertheless, the problem of LTBI remains highly relevant, particularly in the context of post-COVID immune dysregulation, underscoring the need for further improvement in early diagnostic and preventive measures.

## 2. Pathogenic Mechanisms of Latent Tuberculosis Infection

LTBI represents a state of *Mycobacterium tuberculosis* (Mtb) persistence in the host without clinical manifestations of active disease and without bacterial shedding into the environment. The pathogenesis of LTBI is based on a complex interplay between the virulence of Mtb and the host immune response [7].

The persistence of mycobacteria within the human body, which forms the substrate for LTBI, is made possible through several mechanisms, including dormancy, drug tolerance, L-form transformation, and bacterial intercellular communication (the quorum-sensing phenomenon). Consequently, LTBI serves as a continuous reservoir for the potential development of ATB [8].

Early detection of various states of tuberculosis infection has significant implications for improving the epidemiological situation. According to Drain R.K. et al. (2018), these conditions can be viewed as sequential and heterogeneous stages following the initial contact with the infectious source [9]. They include: (1) the elimination stage, during which the pathogen is cleared from the host; (2) the stage of LTBI, in which Mtb persists in a metabolically inactive state; and (3) the early preclinical stage that precedes the onset of clinically manifest disease. Identification of these stages is of great practical importance, as it enables timely implementation of preventive measures—primarily chemoprophylaxis—which substantially reduces the risk of LTBI progression to ATB and decreases the number of new cases. However, existing diagnostic approaches remain limited: at present, no universal method is available to reliably differentiate the stages of tuberculosis infection in individual patients.

Particular attention in contemporary phthisiology is drawn to the problem of LTBI associated with multidrug-resistant (MDR) Mtb strains. Current estimates suggest that approximately 3 out of every 1000 individuals worldwide are infected with MDR Mtb in a latent form [10]. This phenomenon has serious epidemiological implications, as the spread of such forms of LTBI may substantially hinder future global tuberculosis control efforts.

The immune response plays a pivotal role in the establishment and maintenance of LTBI, including cases caused by multidrug-resistant Mtb. In contrast to drug-susceptible strains, the effectiveness of immune control becomes even more critical in MDR tuberculosis, since chemoprophylactic and therapeutic options are markedly limited.

### 2.1. Innate Immunity Cells in Latent Tuberculosis Infection

Innate immune cells play a crucial role in the primary defense against *Mycobacterium tuberculosis* (Mtb). However, Mtb can exploit certain myeloid cell types for its survival and replication within the host, indicating the ambiguous functional role of these cells in tuberculosis pathogenesis.

When comparing NK-cell concentrations in peripheral blood between patients with latent tuberculosis infection (LTBI) and those with active tuberculosis (ATB), significantly higher levels were observed in individuals with LTBI [11]. Notably, NK-cell levels were also elevated in both LTBI and ATB compared with healthy donors. Expression of the activation marker CD69 increased in ATB, whereas in LTBI it did not differ from healthy controls. The authors also reported elevated concentrations of the NKG2D ligand ULBP-1 in peripheral blood of ATB patients. Memory-like NK cells with a CD45R0+ phenotype were increased in peripheral blood of LTBI patients compared with both healthy controls and ATB patients [11]. Another study demonstrated increased expression of the imprinting-associated memory marker NKG2C in LTBI, while ATB patients exhibited enhanced expression of CD57, a marker of advanced NK-cell differentiation [12]. IFN-γ expression was higher in peripheral NK cells in ATB than in healthy controls and LTBI patients [13]. Zhou et al. additionally noted that Mtb-responsive NK cells tend to upregulate KLRG1 during tuberculosis progression [13].

Albayrak et al. reported that the NKp30 and NKp46 markers were more highly expressed in both CD56^−^ and CD56dim NK subsets in ATB, together with increased expression of granzymes A and B. In contrast, LTBI patients exhibited higher expression of late maturation markers (CD57, KIR) in the CD56^−^ subset, as well as greater IFN-γ production following stimulation compared with ATB patients [14]. Collectively, NK cells in LTBI predominantly display a memory-like phenotype and reduced expression of activation markers; nevertheless, upon *in vitro* stimulation they produce greater quantities of effector cytokines than NK cells from ATB patients. This may reflect impaired NK-cell activation quality in ATB.

Macrophages play a central role in granuloma formation during tuberculosis infection. Impairment of their function may contribute to Mtb dissemination. Iswanti et al. demonstrated reduced NADH oxidase activity in macrophages from ATB patients; however, β-glucuronidase, acid phosphatase and myeloperoxidase activity was increased compared with both healthy controls and LTBI patients [15]. Phenotypic analysis of monocytes in LTBI revealed higher expression of CX3CR1 and CD36 compared with healthy donors, as well as an increased proportion of “non-classical” monocytes expressing IL-6 and TNF-α [16]. CD14+CD16+ monocytes were elevated in peripheral blood of ATB patients compared with healthy controls and LTBI patients [17]. Moreover, CD14+CD16– monocytes expressing pro-inflammatory markers S100A9, S100A12, RETN, S100A8, PPBP (CXCL7) and PF4 (CXCL4) were more abundant in ATB than in LTBI. Mubeen et al. observed increased proportions of “intermediate” and “non-classical” monocytes in both LTBI and ATB [18]. Of particular interest are data showing elevated numbers of IL-6-expressing monocytes in LTBI compared with ATB and healthy donors. Current literature also suggests an association between CD16+ monocytes and TB severity [19]. Thus, increased monocyte/macrophage activity and frequency in peripheral blood may reflect reactivation of the tuberculous process.

Dendritic cells (DCs) are essential for antigen presentation, and their normal function is critical for developing an effective adaptive immune response to Mtb. A multi-cohort study found that resting NK cells, activated NK cells, M2 macrophages and eosinophils increased in LTBI, whereas γδ T cells, monocytes, M0 macrophages, activated dendritic cells, activated mast cells and neutrophils were elevated in ATB [20]. Other researchers reported reduced frequencies of plasmacytoid dendritic cells (CD11c−CD123+) and type 2 myeloid dendritic cells (CD11c+CD123−CD141+) in ATB compared with LTBI and healthy controls [21]. These findings are consistent with Parlato et al., who observed reduced proportions of myeloid BDCA3+ and plasmacytoid CD123+ DCs in ATB, along with impaired IFN-α signaling in these cells [22]. ATB DCs were also shown to express increased levels of the immunoinhibitory receptor BTLA [23]. Despite variability in reported circulating DC levels in ATB and LTBI, recent studies collectively suggest functional impairment of DCs in ATB, particularly involving defective presentation of Mtb antigens to adaptive immune cells.

iNKT and MAIT cells provide immune protection at mucosal surfaces, supporting their proposed involvement in early host responses to Mtb. LTBI patients exhibit higher levels of iNKT cells compared with ATB and healthy controls, while iNKT cells in ATB upregulate HLA-DR and PD-1 and downregulate CCR6 [24]. Another study assessing intracellular cytokine expression found that IFN-γ-producing iNKT cells were increased in LTBI, whereas IL-17-producing iNKT cells were reduced compared with healthy donors and ATB patients [25]. Thus, LTBI is characterized by an elevated frequency of IFN-γ-producing iNKT cells, which contribute to granuloma formation and containment of Mtb dissemination.

In ATB, MAIT cells show reduced IFN-γ production, as well as diminished expression of TNF-α, IL-17F, granulysin and granzyme B [26]. Jiang et al. reported similar findings in bronchoalveolar lavage fluid from patients with pulmonary tuberculosis, showing decreased MAIT-cell IFN-γ production [27]. Consistent observations were made in children with ATB, who exhibited reduced frequencies of CD3+CD4−CD161highVα7.2+ MAIT cells in peripheral blood [28]. These cells also displayed an activated phenotype (CD69+) compared with children with LTBI and healthy donors, yet they did not accumulate or proliferate in the lungs during active disease. Overall, ATB is associated with impaired function of both iNKT and MAIT cells, contributing to pathogen dissemination and disease progression.

### 2.2. T Cells in Latent Tuberculosis Infection

The T-cell arm of the adaptive immune response plays a key role in limiting the dissemination of tuberculosis infection. This function is mediated through the formation of granulomas as part of a type 1 immune response [4]. The key cells involved in granuloma formation during Mtb infection are IFN-γ–producing CD4^+^ and CD8^+^ T-lymphocytes that express the transcription factor T-bet, as well as chemokine receptors such as CXCR3 and CCR5 [29]. The study of these T cell populations—both in peripheral blood and at the site of infection—remains an important area of research in tuberculosis immunology. Given the absence of symptoms and bacterial shedding in LTBI, compared with ATB, it can be inferred that the nature of the immune response limiting infection differs substantially between these conditions.

Numerous studies have attempted to characterize T cell phenotypes in LTBI and determine their role in the immunopathological process [30,31,32]. Luo et al. demonstrated that the percentage of T cells in peripheral blood was lower in LTBI than in ATB patients; however, the absolute counts of CD4^+^ and CD8^+^ T cells were significantly higher in LTBI. In contrast, ATB patients exhibited increased expression of HLA-DR on both CD4^+^ and CD8^+^ T cells [30]. Similarly, Qin S. et al. reported that ATB patients had reduced overall levels of CD8^+^ T cells compared with LTBI, but these cells showed elevated expression of effector molecules such as granzyme A, granzyme B, and perforin. Most Mtb-specific CD8^+^ T cells displayed a TEMRA (CD45RA^+^CCR7^−^) phenotype [31]. Another group confirmed that Mtb-specific CD8^+^ T cells in LTBI predominantly exhibit the TEMRA phenotype (CD45RA^+^CCR7^−^) with high surface expression of 2B4 and CD160, whereas in ATB, the predominant phenotype was effector memory (EM; CD45RA^−^CCR7^−^) expressing only 2B4 [32].

Mtb-specific CD4^+^ T cells, the principal cells driving granulomatous inflammation, have also been intensively investigated. Several studies have compared Mtb-specific CD4^+^ T cells in LTBI and pulmonary ATB [33,34]. Arlehamn et al. demonstrated that in LTBI, Mtb-specific CD4^+^ T cells predominantly display a CCR6^+^CXCR3^+^CCR4^−^ phenotype corresponding to Th17.1 cells, which are increased in peripheral blood compared with healthy controls [33]. Moreover, CD4^+^CCR6^+^CXCR3^+^CCR4^−^ T cells exhibit a unique transcriptional profile associated with enhanced T cell activation, increased survival, induction of CTL-like cytotoxicity, and migration to inflammatory sites. These findings were corroborated by Nikitina et al., who reported that IFN-γ^+^ lymphocytes constitute the main subpopulation of Mtb-specific CD4^+^ T cells in the blood of tuberculosis patients [34]. Most of these cells exhibited a CXCR3^+^CCR6^+^ phenotype [34].

Notably, such Mtb-specific CD4^+^CXCR3^+^CCR6^+^ T cells were almost exclusively found within the CD45RA^−^ memory T-helper subset in LTBI patients [35]. Consistent results were obtained by Izumida et al., who observed an increased frequency of Mtb-specific CD4^+^ T cells in LTBI, predominantly displaying central and effector memory phenotypes with high IFN-γ and IL-17 expression. In contrast, ATB patients had a predominance of Mtb-specific CD4^+^ T cells expressing IL-10 and IFN-γ, reflecting a more pronounced regulatory component during active disease [36]. In contrast to LTBI, Mtb-specific CD4^+^ T cells in ATB display a diminished capacity for co-expression of CXCR3 and CCR6 on T helper cells [37].

Cytokine expression profiling of CD4^+^ and CD8^+^ T cells revealed that patients with LTBI had higher levels of IFN-γ, TNF-α, and IL-2 expression within CD4^+^ T-lymphocytes compared with both healthy donors and ATB patients. Notably, cytokine production by CD8^+^ T cells did not differ significantly between LTBI and ATB [38]. These findings suggest a more efficient helper T cell response to Mtb compared to the cytolytic T-cell response. Next, Fang et al. reported that the frequencies of CD4^+^ T cell subsets with IFN-γ^+^CD27^−^, IFN-γ^+^CD38^+^, and IFN-γ^+^CD27^−^CD38^+^ phenotypes were higher in ATB than in LTBI, supporting the hypothesis of T-helper hyperactivation during ATB [39]. Similarly, Caccamo et al. found that triple cytokine expression (IFN-γ, TNF-α, and IL-2) by CD4^+^ T cells was associated with viable bacterial load and was relatively rare among LTBI patients [40]. Thus, it is important to develop new tools to increase the diagnostic accuracy of current immunologic assays, including flow cytometric analysis. Cytometric assays based on Mtb-specific T cells detection have several limitations, including challenges in standardizing the assays for clinical use, relatively high coast, the obtained data does not reflect the totality of Mtb-specific T cell, etc. Furthermore, it is still poorly understood the relationship between the levels of antigen-specific T cells and the assessment of treatment success, the identification of pulmonary and extrapulmonary cases of tuberculosis as well as the infection outcome, but the efforts to develop effective and reasonably priced commercial assays should be underway.

Furthermore, ATB patients exhibit reduced frequencies of IL-17–producing CD3^+^CD161^+^, CD3^+^CD4^+^CD161^+^, and CD3^+^CD8^+^CD161^+^ T cells compared with both healthy controls and LTBI patients [41]. Similar findings were reported by Sharma et al., who observed decreased levels of CD161^+^ CD8^+^TRAV1–2^+^ MAIT cells in ATB relative to LTBI and healthy subjects [42].

Circulating T regulatory (Treg) cells are characterized by the presence of Forkhead box P3 transcription factor (FoxP3), high expression of CD25 (α-chain of IL-2 receptor), and low expression of CD127 (α-chain of IL-7 receptor) on their surface [43]. Tregs are known to modulate the activity of antigen presenting cell, to down-regulate the functions of different types of effector cells, including CD4+ and CD8+ T cell subsets [44]. Interestingly, Luo et al. also reported elevated frequencies of regulatory T cells (Tregs) in the peripheral blood of ATB patients compared with those with LTBI [12]. This may represent a compensatory response to hyperactivation of a less effective peripheral immune response. Semple et al. noticed that in peripheral blood samples from patients with ATB the frequency of CD4^+^CD25^+^FoxP3^+^ Tregs was higher vs. patients with LTBI, and higher levels of Tregs cells were detected in bronchoalveolar lavage (BAL) of patients with TB compared to LTBI [45]. Similarly, Zewdie et al. found that the frequency of CD4^+^CD25^+/hi^ T cells was higher in patients with TB compared to LTBI individuals, and a significant increase in FoxP3 expression in active primed Tregs (CD4^+^CD25^+^FoxP3^+^CD127^lo^CD45RO^+^Ki-67^+^) was also observed in patients with ATB vs. patients with LTBI [46]. Furthermore, the levels of peripheral blood CD4^+^CD25^+^FoxP3^+^ T cells and CD4^+^CD25^+^Foxp3^+^PD-1^+^ T cells were significantly increased from healthy controls to patients with LTBI to patients with ATB, respectively [47]. Finally, it was shown that CD4^+^CD25^high^FoxP3^+^ cells were able to down-modulate in vitro microbicidal activity against Mtb, suggesting that Tregs may suppress the protective immune response against Mtb, leading to pathogen persistence, as well as disease progression during both ATB and LTBI [48]. All these findings suggest that Treg cells are significantly augmented especially in patients with ATB, pointing to a potential role of these cells in disease progression.

Summarily, patients with LTBI had increased frequencies of CD4^+^ and CD8^+^ T cells, but these cell subsets expressed decreased levels of activation antigens and effector molecules then the cells from ATB patients. Next, Mtb-specific CD8^+^ T cells in LTBI predominantly exhibit the TEMRA phenotype (CD45RA^+^CCR7^−^), while Mtb-specific CD4^+^ T cells predominantly displaying central and effector memory phenotypes with high IFN-γ and IL-17 expression and had CCR6^+^CXCR3^+^CCR4^−^ phenotype corresponding to Th17.1. Moreover, patients with LTBI had higher levels of polyfunctional memory (IFN-γ^+^TNF-α^+^IL-2^+^) CD4^+^ T cells if compared with ATB patients. Finally, the frequencies of circulating Treg cells were reduced in LTBI vs. ATB. These data suggest that during tuberculosis infection, heightened peripheral T-cell activation occurs, followed by migration to the site of inflammation—supported by the elevated effector potential and terminal differentiation phenotypes of these cells. However, the effectiveness of this response appears limited, possibly due to high bacterial burden or the induction of T cells with a regulatory phenotype. In contrast, LTBI is characterized by an absence of elevated effector molecule expression in T cells, relative stability of peripheral T cell subsets, and a memory-like phenotype with high CXCR3 and CCR6 expression, suggesting effective migration and local immune control of Mtb within granulomatous lesions.

### 2.3. B Cells in LTBI

Despite the well-established role of the T cell arm of adaptive immunity in the response to Mtb infection, B-lymphocytes are also involved in limiting inflammation and contributing to pathogen elimination. Immunohistochemical analysis of tissue samples from both experimental animals [49,50] and patients infected with Mtb [51] revealed the accumulation of CD19^+^ lymphocytes at the periphery of necrotizing granulomas. Moreover, within the inflammatory foci, structures resembling germinal centers of lymph nodes containing proliferating B lymphocytes were observed [52]. These findings suggest the possible formation of tertiary lymphoid organs within the pathological sites of mycobacterial infection.

Notably, in addition to B cells, granulomas contained CXCR5^+^ T cells—so-called follicular helper T cells (Tfh)—located in close proximity to B cell clusters. An increased level of these Tfh cells has been associated with LTBI, whereas in ATB their higher frequency correlated positively with a favorable outcome of the disease [52].

Dynamic changes in circulating B-lymphocytes have also been observed in LTBI and ATB. In their study, Joosten et al. demonstrated that the frequency of circulating B cells in patients with LTBI and in those receiving anti-tuberculosis therapy did not differ significantly, whereas a reduction in circulating B cells was noted in ATB [53]. Similarly, Chowdhury et al. confirmed these findings and further showed that the proportion of ‘naïve’ B cells in LTBI was decreased in peripheral blood compared with ATB [54]. However, La Manna et al. found a reduced proportion of un-switched memory IgD^+^CD27^+^ B cells in LTBI compared with ATB [55]. It was also noticed that in patients with LTBI the levels of IgM exhibited a significant positive correlation with frequencies of CD21–CD27+ activated and atypical CD21–CD27– memory B cells as well as CD19+CD21–CD20– plasma cells [56]. Next, the circulating levels of IgG were positively associated with CD21–CD27+ activated memory B cells and CD19+CD21–CD20– plasma cells.

The generation of an effective B cell response to Mtb also involves the regulatory component of humoral immunity, represented by regulatory B cells (Breg), which can suppress T cell immune responses. Bregs with the CD19^+^CD24hiCD38hi phenotype have been shown to inhibit CD4^+^ T cell subsets, including Th1 and Th17 cells, both of which play crucial roles in anti-tuberculosis immunity. Additionally, cytokines secreted by Bregs promote the maturation and differentiation of FoxP3^+^CD4^+^ T cells with strong regulatory potential [57]. According to recent studies, patients with ATB exhibit an increased frequency of CD19^+^CD1d^+^CD5^+^ Bregs in peripheral blood [58,59,60]. These cells demonstrate high IL-10–producing capacity and show an inverse correlation with circulating Th17 levels [58]. Krause et al. further demonstrated that CD1d^+^CD5^+^ B-cell concentrations in lung tissue from patients with pulmonary tuberculosis were higher than in paired peripheral blood samples [59]. Conversely, the frequency of CD19^+^CD24hiCD38hiCD5^+^ Bregs was reduced in the peripheral blood of ATB patients compared with LTBI [60], suggesting Breg migration to inflammatory foci and suppression of local immune responses to Mtb. In murine models, IL-35^+^ Bregs were found to infiltrate the spleen, bone marrow, and lung tissue of Mtb-infected mice, with their levels significantly decreasing after effective therapy. Moreover, administration of exogenous IL-35 increased IL-35^+^ Breg levels and inhibited Th1/Th17 differentiation, confirming the suppressive effect of anti-inflammatory cytokines on the development of protective immunity against tuberculosis [61].

Summarily, our current knowledge of the role of B cells in LTBI is limited, yet current data suggest altered functions of B cells during LTBI, linked with the reduction in ‘naïve’ and un-switched memory B cells. It should be noted that differences in the frequency of circulating immune cell subsets between latent and active tuberculosis should be interpreted with caution. Peripheral blood measurements do not fully capture immune dynamics at the primary sites of Mycobacterium tuberculosis infection, particularly the lung and regional lymph nodes. Altered frequencies of antigen-specific CD4^+^ and CD8^+^ T cells in circulation may reflect differential recruitment, tissue retention, or redistribution rather than intrinsic functional superiority or inferiority. Consequently, circulating immune signatures should be regarded as systemic correlates of disease state rather than direct surrogates of protective or pathogenic immune mechanisms.

Next, patients with LTBI exhibited decreased frequencies of different subsets of Breg cells, and the reduced numbers of Bregs may fail to limit excessive inflammatory responses during LTBI. Moreover, increased numbers and activity of Bregs in patients with ATB suggest that B cells are involved in immunopathology during active disease. It is likely that in ATB, hyperactivation of B cells enhances the differentiation of the regulatory arm of the immune system, thereby suppressing the formation of an effective immune response and promoting pathogen dissemination—processes not observed in LTBI. Finally, it will be useful to examine different B cell subsets for their potential use as biomarkers for differential diagnosis of LTBI and ATB.

### 2.4. Extracellular Vesicles in LTBI

Other important players in the pathogenesis of tuberculosis are extracellular vesicles (EVs). These nanosized membrane particles are secreted by almost all types of cells and serve as carriers of various biomolecules, including proteins, lipids, nucleic acids, and metabolites [62]. EVs act as mediators of intercellular communication, influencing both physiological and pathological processes and playing a central role in the pathogenesis of numerous diseases, including infectious ones [63].

As in other infections, tuberculosis involves a bidirectional interaction between the pathogen and the host; therefore, both mycobacterial extracellular vesicles (MEVs) and host-derived vesicles, including those released by immune cells in response to infection, must be considered in the development of tuberculosis infection [64]. EVs derived from the bacterial plasma membrane are enriched with immunomodulatory components that can either enhance or suppress the inflammatory response depending on the stage of disease, as well as virulence factors facilitating bacterial invasion [65]. Conversely, EVs from infected host cells frequently carry mycobacterial antigens and signaling molecules that potentiate innate and adaptive immune responses [66,67].

During ATB, Mtb actively produces EVs in response to various factors such as iron availability, the vesiculogenesis and immune response regulator protein VirR, and the SenX3-RegX3 system, under stress conditions including antibiotic exposure and nutrient deprivation [68,69,70]. These vesicles contain more than 200 proteins, including virulence-associated proteins (ClpB, SodB, EphG, and HspX), lipoproteins (LpqH, LprA, LppX, PstS1, and LprG), and lipids such as phosphatidylinositol dimannoside (PIM) and lipoarabinomannan (LAM) [64,71,72,73]. This cargo, acting as pathogen-associated molecular patterns (PAMPs), activates Toll-like receptor 2 (TLR2) and triggers cascades leading to the production of pro-inflammatory cytokines (IL-1β, IL-6, TNF, IL-12, CXCL1, MIP-1α/CCL3), as well as the anti-inflammatory cytokine IL-10 in macrophages [71,74].

In addition, EVs derived from Mtb cultures increase the expression of major histocompatibility complex molecules (MHC-I and MHC-II) and co-stimulatory molecules (CD86) in dendritic cells, thereby promoting their maturation and antigen presentation [75]. Conversely, LAM present in MEVs can inhibit T-cell maturation following antigen-presenting cell interaction by suppressing IL-2 production [76]. Furthermore, prolonged activation of TLR2 may reduce MHC-II expression and antigen presentation to T cells, promoting mycobacterial survival and persistence [77].

EVs derived from infected host cells possess immunomodulatory properties essential for protection against infection. Such vesicles can activate macrophages and neutrophils, promote bacterial killing, and induce activation of endothelial cells, facilitating leukocyte migration to infection sites [66]. EVs from infected neutrophils have been shown to induce dendritic cell maturation and activate antigen-specific Th1 responses [78]. These effects are mediated by the molecular cargo packaged within the vesicles. For instance, host-derived EVs isolated from the serum of patients with ATB carry peptides from 33 unique mycobacterial proteins (e.g., Cfp2, Mpt32, Mpt64) essential for bacterial adhesion and intracellular survival [79]. The diagnostic panel developed using mass spectrometry increased detection rates of tuberculosis up to 90% among patients with both pulmonary and extrapulmonary disease [79].

Lipidomic analysis revealed decreased levels of certain lipids, such as free fatty acid (FFA 20:1), in urinary EVs from patients with ATB, which distinguished them from both healthy donors and LTBI patients [80]. MicroRNAs (miRNAs) have also emerged as potential diagnostic markers; elevated levels of several miRNAs (e.g., miR-576-3p, miR-483-5p, miR-144) have been identified in plasma-derived EVs from tuberculosis patients [81,82,83]. Lu et al. detected small RNAs (ASdes and MTB-miR5) exclusively in serum EVs from patients with ATB, but not in those with community-acquired pneumonia or in healthy controls [84].

Long noncoding RNAs (lncRNAs) within EVs also represent potential biomarkers. Fang et al. demonstrated reduced expression of NONHSAT101518.2, NONHSAT067134.2, NONHSAT148822.1, and NONHSAT078957.2 in plasma EVs from tuberculosis patients compared with healthy individuals [85]. Of particular interest is miR-20b-5p, which regulates apoptosis in infected macrophages. Mtb downregulates miR-20b-5p expression, while infected macrophages release exosomes containing this miRNA [86]. Reduced miR-20b-5p expression promotes bacterial survival by inhibiting macrophage apoptosis through upregulation of Mcl-1, suggesting miR-20b-5p as a potential therapeutic target in tuberculosis [86].

In LTBI, Mtb enter a dormant, non-replicating state within granulomas, and the composition of EVs reflects this metabolic shift. The peptide of glutamine synthetase (GlnA1) was detected in 82% of LTBI patients, suggesting that Mtb proteins involved in nitrogen metabolism may serve as candidate biomarkers for LTBI detection [87]. Additional potential markers include GarA, chaperones GroES and DnaK, and fatty acid synthase (AcpM), identified in 16% of patients [87]. EVs isolated from urine of LTBI patients showed elevated levels of lipids, including FFAs, diacylglycerols (DAGs), and monoacylglycerols (MAGs). Among these, FFA (20:1) demonstrated the greatest potential for distinguishing LTBI from both healthy individuals and ATB patients [80]. Unlike ATB, EVs in LTBI appear to maintain immune homeostasis, although persistent signaling via TLR2 may suppress T cell function, contributing to long-term bacterial persistence [77,87].

The clinical potential of EVs is highly promising and covers both diagnostic and therapeutic applications. Advantages include their noninvasive collection, stability in biological fluids such as serum and urine, and applicability in patients with immunosuppression or extrapulmonary disease [79,88,89,90]. As mentioned above, various components of EVs, including proteins, lipids, and miRNAs, could serve as potential diagnostic tools with high performance for detecting LTBI, offering detection rates up to 95%, AUC values exceeding 0.95 for differentiation from ATB, and 100% specificity in certain lipid profiles [66,80,91]. Moreover, the integration of multiple approaches markedly enhances diagnostic performance. For example, an enzyme-linked immunosorbent assay (ELISA) combined with dark-field microscopy targeting mycobacterial virulence factors (lipoarabinomannan and its carrier protein) in EVs proved effective for diagnosing tuberculosis in children [90].

Therapeutically, EVs serve as versatile platforms for TB vaccines and drug delivery, helping to overcome multidrug resistance and limited tissue penetration. Several studies have demonstrated that EVs derived from infected macrophages exhibit immunogenicity in vitro and in vivo, inducing protective CD4^+^ and CD8^+^ T cell responses and Th1 polarization [92,93]. Compared with BCG vaccination, this strategy achieved superior efficacy in murine models, with significantly lower Mtb loads in the lungs and spleen [93]. Computational modeling has also identified mycobacterial proteins (DnaK, GrpE, LpqH, HBHA, LprA, LprG, and MPT83) as potential peptide vaccine candidates, with the TLR4 agonist (RpfE) proposed as an adjuvant [94]. For targeted drug delivery, vesicles modified with the peptide Angiopep-2 have been designed to deliver rifampicin across the blood–brain barrier for the treatment of central nervous system tuberculosis [95]. EVs are currently being explored as adjuncts to standard anti-tuberculosis regimens. For instance, vitamin C-pretreated exosomes could kill *M. bovis* BCG in vitro via ROS induction [96]. Furthermore, EVs derived from mesenchymal stem cells, when used alongside standard therapy, improved anti-inflammatory and antimicrobial effects in murine models of renal tuberculosis [97].

Despite their considerable potential, the clinical use of EVs for tuberculosis diagnosis and treatment is hindered by several major challenges, most of which relate to manufacturing and quality control. Current isolation methods, such as differential ultracentrifugation, often produce inconsistent batches with low yield and purity, limiting scalability and diagnostic reliability [98]. Functional characterization and standardization remain insufficient: variability in protein cargo, size, and potency and possible contamination can reduce efficacy and cause safety risks [67]. Another major challenge is the development of a reproducible and reliable dosing method [99]. The most commonly used dosing methods (particle number and total protein quantification) exhibit considerable inter- and intra-laboratory variability, and a standardized understanding of EV pharmacokinetics remains lacking [99]. These challenges highlight the urgent need for specific guidelines, based on established frameworks such as MISEV, to ensure standardized, safe, and effective translation of EVs into routine clinical practice [100].

## 3. Preclinical Stage of Latent Tuberculosis Infection (LTBI)

The preclinical stage of LTBI is associated with the activation of both innate and adaptive immune responses [9,101]. Schenkel et al. have demonstrated enhanced production of IFN-γ, TNF-α, and IL-2, as well as the emergence of polyfunctional T-lymphocytes, reflecting the activation of antimycobacterial defense mechanisms [102]. Quantitative parameters of Interferon-Gamma Release Assays (IGRAs) correlate with the risk of disease progression, although their prognostic value remains limited [103]. A promising direction involves the assessment of T cell exhaustion markers such as PD-1 and TIM-3, which are associated with infection reactivation [104].

### 3.1. Transcriptomic and Metabolomic Studies

The application of “omics” technologies has enabled the identification of transcriptomic signatures that predict the development of ATB. Interferon-associated gene signatures can forecast disease progression 6–12 months prior to clinical manifestation [105,106]. Weiner and Kaufmann revealed alterations in lipid and energy metabolism among individuals with LTBI, reflecting host adaptation to the chronic persistence of Mtb [107].

### 3.2. Radiological Investigations

Functional imaging techniques such as 18F-FDG PET/CT have demonstrated high sensitivity in detecting subclinical disease activity. Among asymptomatic individuals with positive immunological tests, metabolically active foci in the lungs have been identified, serving as predictors of LTBI progression [108].

### 3.3. Experimental Models

The rhesus macaque model of latent infection has shown that in some animals LTBI persists for extended periods, whereas in others it progresses to active disease. These models allow for the identification of immunological risk markers and the testing of novel vaccine strategies [109].

The results of preclinical LTBI studies have practical significance for:

- *Personalized prevention:* identifying individuals at high risk of progression and implementing targeted chemoprophylaxis;
- *Biomarker development:* enabling early diagnosis of subclinical disease activity; (chemokine induced by IFN-γ; TB27.4; CD27 expression on Mtb-specific CD4^+^ T cells et);
- *Vaccine innovation:* designing new immunomodulatory and vaccination approaches to prevent infection reactivation.

Collectively, preclinical LTBI research indicates that this is a dynamic condition accompanied by complex alterations in immune function and metabolism (Table 1). The integrated use of immunological, transcriptomic, and radiological methods provides new opportunities for predicting the risk of ATB and refining preventive strategies [110].

## 4. Host-Related Modifiers of LTBI Immune Responses

Host factors such as age, HIV status, diabetes mellitus and nutritional status are recognized modulators of the immune response to M. tuberculosis, and they may influence both susceptibility to LTBI and the performance of immunological assays. Older age has been associated with reduced T-cell responsiveness and lower IFN-γ release, potentially decreasing the sensitivity of IGRAs. HIV infection alters CD4^+^ T-cell-dependent immune pathways, which can result in indeterminate IGRA results and atypical cytokine responses. Diabetes and protein–energy malnutrition are likewise associated with impaired cell-mediated immunity, reduced polyfunctional T-cell responses and altered inflammatory signatures. Although these factors were not individually stratified in most of the included studies, they represent important sources of heterogeneity and should be considered when interpreting LTBI biomarkers [114].

Most of the studies included in this review were conducted in Russia or European settings, which limits the generalizability of the findings to regions with the highest LTBI burden, particularly Africa and Asia. Populations in high-incidence settings may exhibit distinct immunological phenotypes due to repeated exposure to *M. tuberculosis*, different BCG vaccination strategies, environmental mycobacteria prevalence and co-endemic infections such as HIV or helminths. These contextual factors can reshape innate and adaptive immune responses, potentially modifying the performance and predictive value of candidate biomarkers. The lack of proportional representation from high-burden regions therefore constitutes a limitation of the present evidence base [115,116].

Children exhibit qualitatively different immune responses compared with adults, including reduced Th1 polarization, distinct cytokine kinetics and age-dependent maturation of innate immunity. These differences may affect both the trajectory of LTBI and the performance of diagnostic assays such as IGRAs, which tend to have lower sensitivity in young children. Despite the clinical importance of pediatric LTBI, only limited data in this population were available among the included studies, and pediatric-specific biomarkers remain insufficiently characterized. Future research should specifically address these developmental and immunological differences to improve diagnostic accuracy in children [117,118].

## 5. Conclusions

The preclinical stage of (LTBI) is defined as a biologically active but clinically asymptomatic condition characterized by specific immunological and metabolic alterations. The presented approaches—including immunological assays (IGRA, multiplex T-cell assays, TAM), transcriptomic (“interferon” signatures and others), functional (MGIA), and radiological (PET/CT) methods—demonstrate prognostic potential for identifying individuals at increased risk of progression to ATB [119].

The highest predictive accuracy is achieved through a combined approach, integrating quantitative immune assays and transcriptomic signatures with targeted imaging, which enhances the precision of risk stratification. Experimental models, particularly non-human primates, reproduce the full spectrum of latency and reactivation, providing a platform for preclinical validation of biomarkers and vaccine or immunomodulatory strategies.

At the same time, studies on LTBI associated with MDR-TB are of special relevance. For MDR-LTBI, standard preventive regimens are largely ineffective, making immune surveillance and early detection of subclinical activity critically important.

The preclinical stage of LTBI represents a pivotal link in tuberculosis control, with both theoretical and practical implications for reducing disease burden. Advances in transcriptomics, functional immune testing, and molecular imaging offer real opportunities for targeted prevention and personalized intervention. However, translation from research-derived signatures to clinically applicable, standardized, and cost-effective diagnostic algorithms requires coordinated, multidisciplinary efforts—including large prospective studies, methodological standardization, technology transfer into accessible platforms, and parallel assessment of feasibility and cost-effectiveness.

In practical terms, a future implementation pathway may include early biomarker-based screening, followed by risk-adapted preventive therapy or monitoring, aimed at reducing reactivation rates and limiting transmission of both drug-sensitive and drug-resistant Mtb strains. Achieving this goal will require international collaboration, sustained scientific support, and political commitment to integrate evidence-based strategies into public health programs.

## Figures and Tables

**Table 1:** Methods and their characteristics.

Method/Approach	Principle	Advantages	Limitations	Prognostic Value/Application
Tuberculin Skin Test (TST/PPD)	Delayed-type hypersensitivity—skin reaction to PPD	Accessibility, low cost, long history of use	Cross-reactivity with BCG and NTM; low specificity; no quantitative assessment of immune response	Useful for large-scale screening and contact identification; limited for predicting subclinical activation
TBST (Mtb antigen-based skin tests) Cy-Tb (India); Diaskintest (Russia); C-TST (formerly known as ESAT6-CFP10 test, China)	Delayed-type hypersensitivity—skin reaction to Mtb antigen ESAT-6, CFP-10	Accessibility, low cost	Higher specificity compared to TST; No cross-reactivity with BCG and NTM; no quantitative assessment of immune response	Useful for large-scale screening and contact identification; limited for predicting subclinical activation [1,2,101]
IGRA (QuantiFERON, T-SPOT.TB, TigaTest^®^TB (Russia)	Induction of IFN-γ secretion by T cells upon stimulation with Mtb-specific antigens	Higher specificity compared to TST; quantitative data	Variable results; limited ability to distinguish latency from recent infection; reduced sensitivity in immunocompromised individuals	Elevated IFN-γ levels and dynamic response increases are associated with a higher risk of progression (see Pai et al., 2014) [103]
Quantitative/Multiplex T-cell Assays (Multicytokine, Polyfunctional T cells)	Evaluation of multiple cytokines (IFN-γ, IL-2, TNF-α) and T-cell functionality	Provides a more detailed immunological “landscape”; detects polyfunctional and exhausted phenotypes	Requires laboratory infrastructure; limited standardization	Emergence of polyfunctional T cells and changes in IL-2/IFN-γ ratios may predict progression risk
T-cell Activation Marker (TAM) Assays (CD38, HLA-DR, Ki-67 on Mtb-specific T cells)	Detection of activation/proliferation markers on antigen-specific T cells	High specificity for active or progressing infection in some studies	Requires multicolor flow cytometry and standardized protocols	Useful for identifying individuals in the subclinical phase; studies demonstrate correlation with progression
Transcriptomic Signatures (RNA-seq, Gene Panels)	Assessment of gene expression profiles (e.g., interferon-inducible signatures)	High prognostic value in several cohorts; can detect risk 6–12 months before disease onset	Expensive; requires validation across populations; regulatory and standardization challenges	Zak et al. [105] and Suliman et al. [106] demonstrated predictive potential—promising for targeted prevention
Proteomics/Metabolomics	Identification of protein and metabolic biomarkers in blood/serum	Integrates immune and metabolic alterations; provides additional risk biomarkers	Methodologically complex; requires large-scale validation	May complement transcriptomic signatures to improve predictive accuracy
PET/CT (18F-FDG)	Visualization of metabolically active foci in lungs and lymph nodes	High sensitivity for subclinical lesions; localization of metabolically active sites	Expensive; radiation exposure; not specific for Mtb.	Detection of FDG-avid foci in asymptomatic individuals correlates with high risk of progression [111]
In vitro Mycobacterial Growth Inhibition Assay (MGIA)	Evaluation of host blood/cells’ ability to restrict Mtb growth in vitro	Functional assessment of host antibacterial activity	Requires standardization; not widely implemented	Potentially useful for evaluating individual resistance to progression; applicable in vaccine studies [112]
Bacteriological/Molecular (PCR)	Direct detection of Mtb DNA/RNA (chemokine induced by IFN-γ; TB27.4; CD27 expression on Mtb-specific CD4^+^ T cells, MicroRNA profiles (e.g., miR-155, miR-223) et)	High sensitivity in active disease	In latent infection, culture/PCR results are typically negative—limited value for LTBI	Not suitable for LTBI confirmation; may be used to detect early transition to active disease (biopsy, BAL) [110]
Animal Models (Macaques, Mice) and Experimental Studies	Investigation of latency and reactivation dynamics; testing of interventions	Enable mechanistic insights and hypothesis testing	Limited translational relevance; ethical and resource constraints	Essential for preclinical evaluation of vaccines and immunomodulators [113]

## Data Availability

Not applicable.
